# Coexistence of metamagnetism and slow relaxation of magnetization in ammonium hexafluoridorhenate†

**DOI:** 10.1039/d0ra10325j

**Published:** 2021-02-03

**Authors:** James Louis-Jean, Samundeeswari M. Balasekaran, Keith V. Lawler, Adrián Sanchis-Perucho, José Martínez-Lillo, Dean Smith, Paul M. Forster, Ashkan Salamat, Frederic Poineau

**Affiliations:** Department of Chemistry and Biochemistry, University of Nevada Las Vegas 4505 South Maryland Parkway Las Vegas Nevada 89154-4003 USA louisjea@unlv.nevada.edu poineauf@unlv.nevada.edu; Instituto de Ciencia Molecular (ICMol), Departament de Química Inorgànica, Universitat de València Catedrático José Beltrán 2 46980 Paterna València Spain f.jose.martinez@uv.es; Department of Physics and Astronomy, University of Nevada Las Vegas 4505 South Maryland Parkway Las Vegas Nevada 89154-4003 USA; HPCAT, X-ray Science Division, Argonne National Laboratory Lemont IL 60439 USA

## Abstract

The (NH_4_)_2_[ReF_6_] (1) salt was studied by X-ray diffraction, Raman spectroscopy, theoretical calculations, and magnetic measurements. 1 crystallizes in the trigonal space group *P*3̄*m*1 (Re–F = 1.958(5) Å). In the Raman spectrum of 1, splitting of the observed peaks was observed and correlated to the valence frequencies of vibration of the [ReF_6_]^2−^ anion. The study of the magnetic properties of 1, through DC and AC magnetic susceptibility measurements, reveals the coexistence of metamagnetism and slow relaxation of magnetization at low temperature, which is unusual in the molecular systems based on the paramagnetic 5d metal ions reported so far.

## Abstract 2

Unusual magnetic behaviour of 5d metal ions as studied in (NH_4_)_2_[Re^(IV)^F_6_].

## Graphical abstract

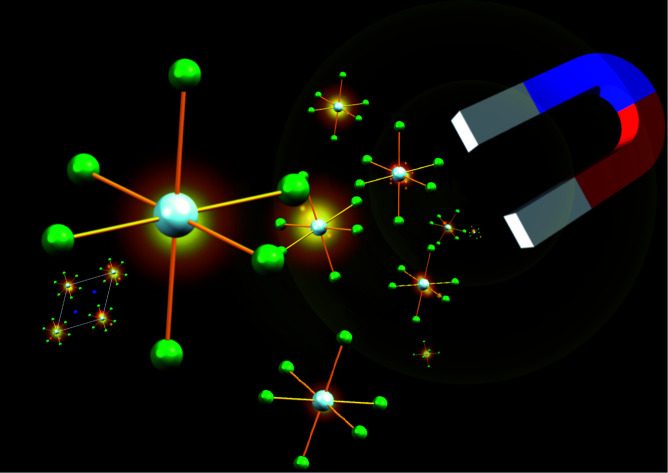

## Introduction

Salts of the [ReX_6_]^2−^ (X = Cl, Br, I) anions have been known for decades and have been spectroscopically and structurally studied.^1–3^ However, the chemistry of the fluorido anion {*i.e.*, [ReF_6_]^2−^} is not as well investigated; this matter of fact is probably due to the difficulty of preparing a [ReF_6_]^2−^ precursor. Hexafluoridorhenate salts can be prepared from the solid-state melting reaction (SSMR) of A_2_[ReX_6_] (X = Cl, Br, I) salts with molten AHF_2_ (A = NH_4_^+^, K^+^). However, the isolation of the subsequent water-soluble A_2_[ReF_6_] salt has shown to be challenging.^4,5^ Recently, detailed procedures for the preparation of K_2_[ReF_6_]^6^ and (NH_4_)_2_[ReF_6_]^7^ have been reported. Currently, only eleven compounds containing the [ReF_6_]^2−^ anion have been characterized by single crystal X-ray diffraction: K_2_[ReF_6_],^6,8^ A_2_[ReF_6_] (A = Rb, Cs),^6^ (AsPh_4_)_2_[ReF_6_]·2H_2_O, (PPh_4_)_2_[ReF_6_]·2H_2_O (Ph = C_6_H_5_: tetraphenylphosphonium),^9,10^ [M(viz)_4_(ReF_6_)]_∞_ (M = Zn, Ni; viz = C_5_H_6_N_3_: 1-vinylimidazole),^9^ [Co_3_(dpa)_4_][ReF_6_]·2DMF (DMF = HCON(CH_3_)_2_ and dpa = C_10_H_8_N_3_: 2,2′-dipyridylamine anion),^11^ (PPh_4_)_2_[ReF_6_]·CH_3_CN,^11^ (BEDO)_4_[ReF_6_]·6H_2_O (BEDO = C_10_H_8_O_4_S_4_: bis(ethylenedioxo)tetrathiafulvalene)^12^ and [Co(NH_3_)_6_]_3_[ReO_4_][ReF_6_]_4_·6H_2_O.^13^

The (NH_4_)_2_[ReF_6_] salt was initially reported^4^ in 1956. While (NH_4_)_2_[ReF_6_] has been studied by X-ray absorption spectroscopy,^7^ thermal gravimetric analysis^14,15^ and was used as a precursor for preparing materials with remarkable magnetic properties,^9,11,12^ up to now there is no available single crystal X-ray data of the salt. In salts containing the [ReX_6_]^2−^ (X = F, Cl, Br, I) anions, the Re(iv) ion (5d^3^) forms a hexakis-monodentate coordination with the halogen atoms resulting in either a regular or slightly distorted octahedral geometry. Studies on the magnetic behavior of [ReX_6_]^2−^ species have shown the existence of strong antiferromagnetic interactions.^16^ The nature of such magnetic interaction occurred through space based on the Re–X⋯X–Re contacts and the size of the halogen. The magnetic properties of several species containing [ReX_6_]^2−^ (X = Cl, Br, I) anions have been studied for more than 65 years.^17–25^ However, species containing the [ReF_6_]^2−^ anion are underrepresented. To our knowledge, only six species of [ReF_6_]^2−^ anion have been magnetically studied, namely K_2_[ReF_6_],^21^ (PPh_4_)_2_[ReF_6_]·2H_2_O (Ph = C_6_H_5_), [M(viz)_4_(ReF_6_)]_∞_ (M = Zn, Ni; viz = C_5_H_6_N_3_),^9^ [Co_3_(dpa)_4_][ReF_6_]·2DMF,^11^ and (BEDO)_4_[ReF_6_]·6H_2_O (BEDO = C_10_H_8_O_4_S_4_).^12^

Overall, there is a need to further develop and explore the structural and magnetic chemistry of hexafluoridorhenate(iv) compounds. In this context, the (NH_4_)_2_[ReF_6_] salt was prepared and characterized by X-ray diffraction (single crystal and powder), IR and Raman spectroscopies, DC and AC magnetic susceptibility measurements, and computational methods.

## Results and discussion

### Preparation of (NH_4_)_2_[ReF_6_] (1)

**Fig. 1:**
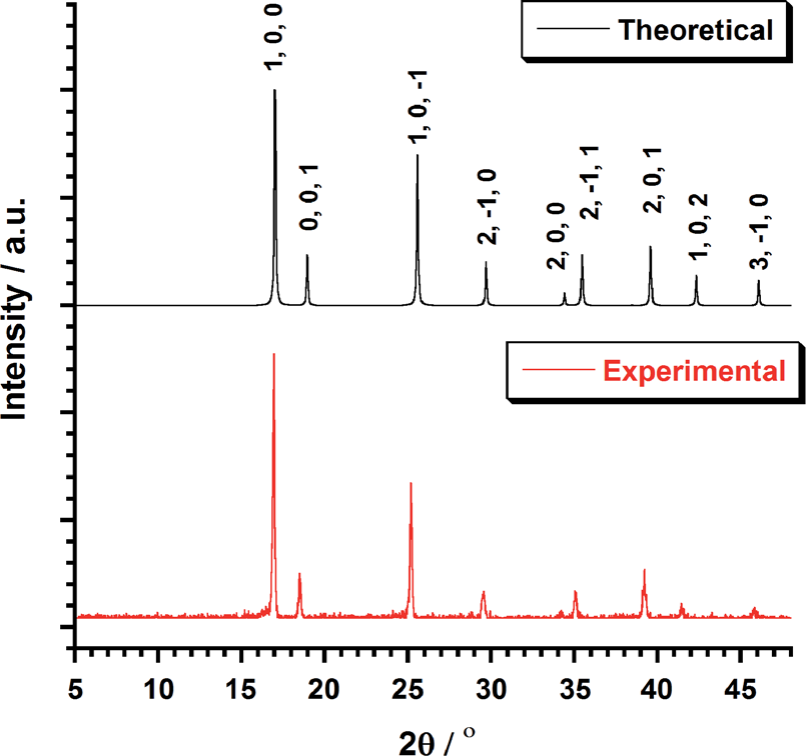
Plot of the theoretical and experimental XRD patterns profile (2*θ*/°) in the range 5–50° for compound 1.

Bifluoride salts, AHF_2_ (A = NH_4_^+^, K^+^) are strong fluorinating agents and have been used for the preparation of [MF_6_]^2−^ (M = Tc, Re) salts.^4–6,9,26,27^ Typically, those preparation involved the reactions of A_2_[MX_6_] salts (X = Cl, Br, I) in molten bifluorides. The isolation of the resulting A_2_[MF_6_] salts is followed by an aqueous workup. This method has been employed unsuccessfully by Peacock^4^ in an effort to prepare 1. Recently, an optimized method for the preparation of A_2_[ReX_6_] salts has been developed from the solid-state melting reaction of (NH_4_)_2_[ReX_6_] (X = Cl, Br) with excess NH_4_HF_2_.^7,9,27^ Using this method, 1 was prepared and separated after washing with a water–methanol mixture. After centrifugation and precipitation, the pink compound was obtained in 60% yield. Colorless single crystals of 1 could be obtained by recrystallization in water or in aqueous HF. The purity and homogeneity of the bulk sample of 1 was confirmed by EXAFS^7^ spectroscopy and powder XRD. The XRD pattern (Fig. 1) shows a single crystalline phase of 1.

### Structure description of 1

**Fig. 2:**
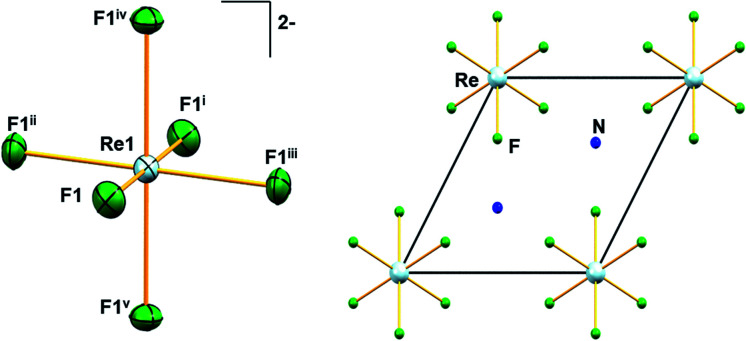
Molecular structure of [ReF_6_]^2−^ anion in 1 showing displacement ellipsoids drawn at 50% probability level (left). Unit-cell plot of 1 along the crystallographic *c*-axis (right). Colour of atoms: Re in light-blue, N in dark-blue and F in green. Symmetry codes: (i) −*x*, −*y*, −*z*; (ii) *x* − *y*, *x*, −*z*; (iii) −*x* + *y*, −*x*, *z*; (iv) *y*, −*x* + *y*, −*z*; (v) −*y*, *x* − *y*, *z*.

Similar to A_2_[MF_6_] (A = K, Rb, Cs; M = Tc, Re) and (NH_4_)_2_[TcF_6_] salts, 1 crystallizes in the trigonal space group *P*3̄*m*1.^6,28^ The structure of 1 consists of [NH_4_]^+^ cations and [ReF_6_]^2−^ anions. The positions of the hydrogen atoms in 1 could not be determined reliably. Thus, the H atoms have not been considered during the refinement procedure. The overall solid-state structure is stabilized by a series of hydrogen bonding. In the [ReF_6_]^2−^ anions, each Re(iv) ion forms a hexakis-monodentate coordination with fluorine atoms (Fig. 2). The Re(iv) ion is located at the origin of the trigonal unit cell occupying the site symmetry, 3̄*m* (Wyckoff position 1*a*) whereas the F atom occupies the m site symmetry (Wyckoff position 6*i*). The six symmetry-related fluorine ligands in the anion result in a regular octahedral geometry (Fig. 2).

All Re–F bond lengths (1.958(5) Å) in 1 are of equal distances and are longer than the Tc–F bond length (1.922(6) Å) in (NH_4_)_2_[TcF_6_].^28^ The Re–F bond length in 1 is also in agreement (Table 1) with other species containing the [ReF_6_]^2−^ anion. In [ReX_6_]^2−^ (X = F, Cl, Br, I) salts, the Re–X bond length generally increases with increasing size of X, 1.958 Å, 2.353 Å, 2.502 Å and 2.721 Å respectively, as expected.^6,16^ DFT bonding analysis of the [ReF_6_]^2−^ anion in its experimental geometry shows the presence of Re–F σ bonds. These σ bonds are nearly ionic with an 85.7% spin-up and 88.8% spin-down occupation on the F atom. The DFT optimized structure of a gas phase (*in vacuo*) [ReF_6_]^2−^ anion is fully octahedral with the Re–F bond lengths extending to 2.004 Å. Bonding analysis provides a nearly identical description of the Re–F bonds in the optimized gas phase anion, indicating that the shorter observed bond lengths are due to packing forces.

**Table 1:** Average Re–F bond lengths (Å) in species containing [ReF_6_]^2−^ as determined by single crystal X-ray diffraction and XAFS spectroscopy (in italic)

Species	Re–F	Ref.
(NH_4_)_2_[ReF_6_]	1.958(5)	This work
	*1.95(2)*	7
K_2_[ReF_6_]	1.948(3)	6
	1.953(4)	8
Rb_2_[ReF_6_]	1.945(7)	6
Cs_2_[ReF_6_]	1.9594(18)	6
[Co(NH_3_)_6_]_3_[ReO_4_][ReF_6_]_4_·6H_2_O	1.942(25)	13
(C_5_H_6_N_2_P)_2_[ReF_6_]·2H_2_O	1.961(7)	9
[Zn(C_5_H_6_N_3_)_4_(ReF_6_)]_∞_	1.957(3)	9
[Ni(C_5_H_6_N_3_)_4_(ReF_6_)]_∞_	1.956(16)	9

**Fig. 3:**
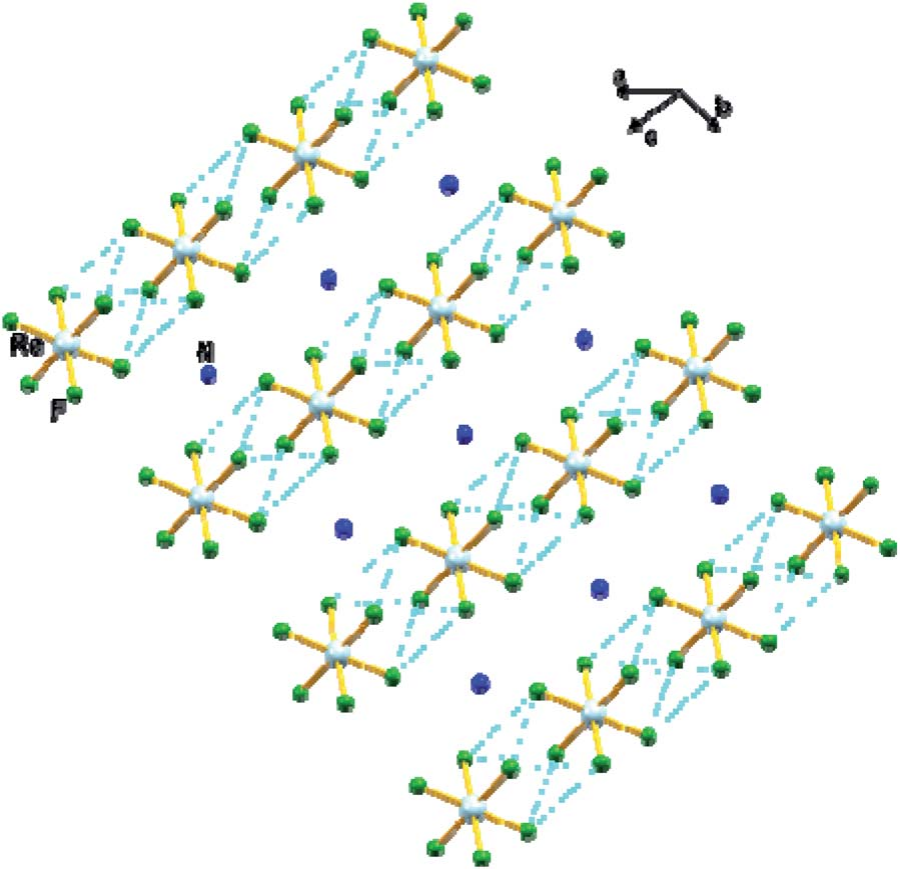
View of a fragment of the crystal packing of 1 showing intermolecular F⋯F contacts *via* Re–F⋯F–Re (dashed aqua line). Colour of atoms: Re in light-blue, N in dark-blue and F in green.

**Fig. 4:**
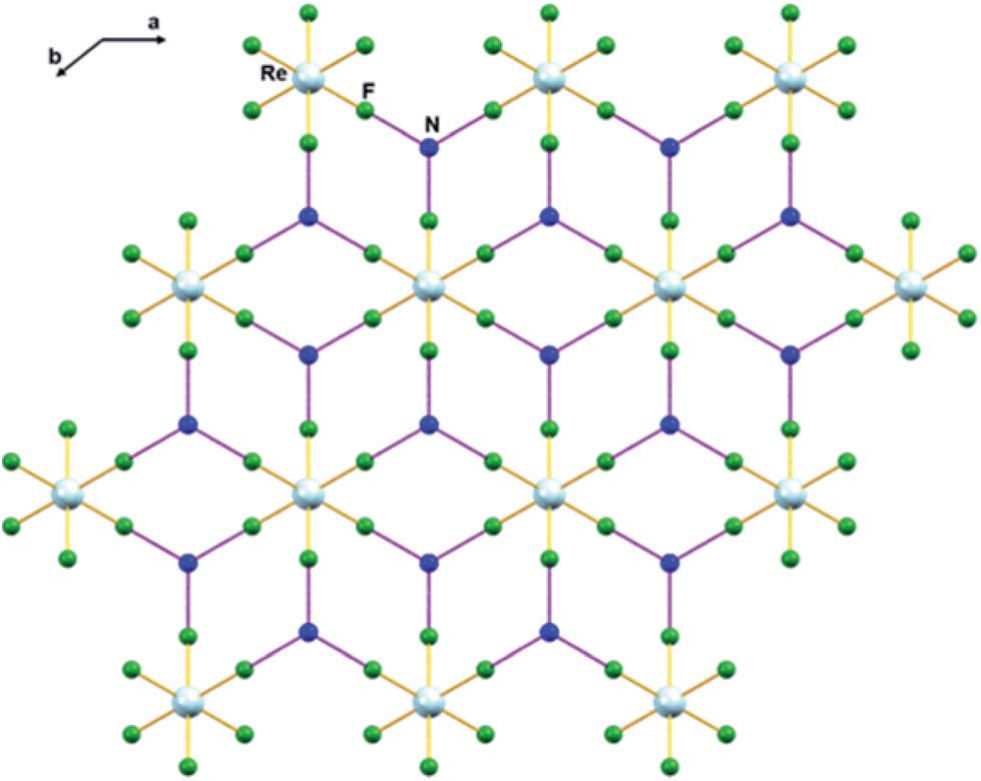
Intermolecular N⋯F interaction in 1*via* Re–F⋯N⋯F–Re (dashed purple line). Colour of atoms: Re in light-blue, N in dark-blue and F in green.

In 1, the shortest inter-ionic Re–F⋯F–Re contact of 2.988(6) Å generates chains of [ReF_6_]^2−^ anions (Fig. 3). This weak intermolecular interaction is expected to be responsible for the occurrence of magnetic interaction between the 5d^3^, paramagnetic Re(iv) atom centers; as seen in the series of [ReX_6_]^2−^ (X = Cl, Br, I) salts.^16,19^ In the tetrahedral [NH_4_]^+^ cation, the short N⋯F contact of the cation and anion, 2.873 Å is responsible for the very weak hydrogen bonds and contributes (Fig. 4) to stabilizing the crystal structure of (NH_4_)_2_[ReF_6_] through a network of Re–F⋯N⋯F–Re contacts.

Further theoretical calculations indicate that there are 4 configurations of the hydrogen atoms of 1 within the *P*3̄*m*1 space group. They differ by whether the H atoms are staggered or eclipsed to the F atoms when viewed along [001] and if the apical H atoms of the [NH_4_]^+^ tetrahedra point towards (001) or (002). Plane-wave DFT optimizations of the atomic positions with the lattice fixed at the experimental value identifies the configuration with staggered H atoms and apical H atoms pointing towards (001) as the most favorable arrangement. The fractional coordinates of the [NH_4_]^+^ atoms in the DFT optimized asymmetric unit cell are N (2/3, 1/3, 0.6903), H1 (2/3, 1/3, 0.9102), and H2 (0.7594, 0.2406, 0.6132). The Re–F bonds are 1.967 Å in reasonable agreement with experiment, and the shortest H⋯F distance is 1.97 Å.

Harmonic vibrational analysis indicates that each of the 4 *P*3̄*m*1 H configurations is dynamically unstable. The displacements of the unstable modes transform the configuration into the one described above or rotate the [NH_4_]^+^ tetrahedra lowering the symmetry to *P*3̄. However, all of those structures are also dynamically unstable. Following the displacements of the *P*3̄ instabilities or alternate initial placements of the H atoms result in lower energy triclinic structures with distorted [ReX_6_]^2−^ octahedra. These distortions shorten some H⋯F distances to emphasize hydrogen bonding. Orientational disorder can occur in ammonium salts, and the disorder increases with temperature potentially changing the structure and Raman spectrum.^29–31^ While the energetic ordering of the disordered structures appears unphysical as they would have been more apparent by XRD, they do indicate that sufficient thermalization of 1 will lead to dynamic distortions that emphasize hydrogen bonding.

### Raman spectroscopy of 1

In the alkali salts of [MF_6_]^2−^ (M = Tc, Re) where the anions are compressed along the crystallographic *c*-axis, the molecular symmetry of the [MF_6_]^2−^ is lowered from *O*_h_ to *D*_3d_. The correlation between the effects of symmetry lowering and the vibrational spectra of the alkali salts of [MF_6_]^2−^ (M = Tc, Re) are well studied.^6,28,32,33^ The slight increase of M–F bond lengths, moving from K_2_[MF_6_] to Cs_2_[MF_6_], as well as the different degrees of symmetry lowering lead the Raman bands to shift to lower frequencies.

**Fig. 5:**
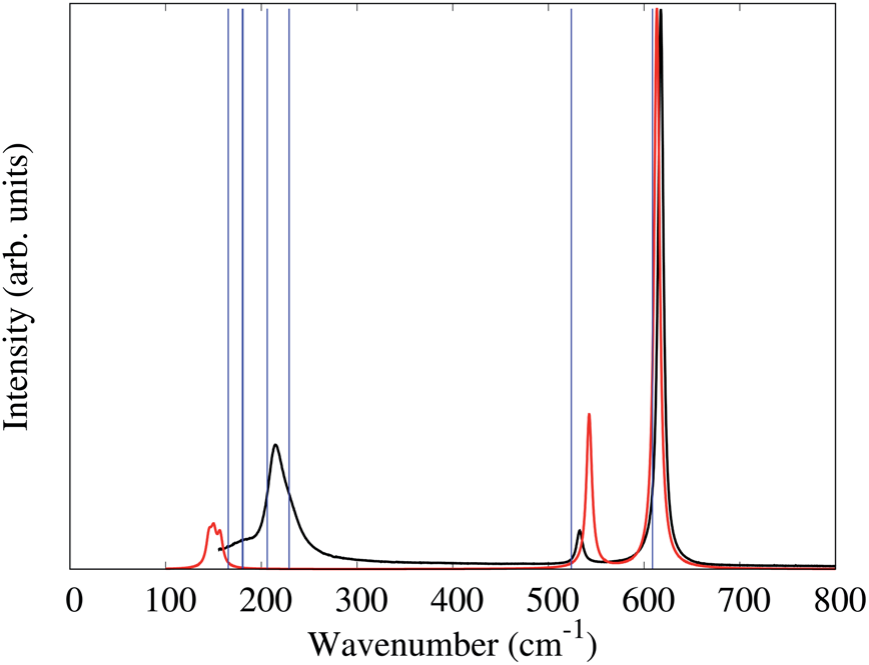
The measured Raman spectrum of 1 (black). The predicted spectrum for a gas phase [ReF_6_]^2−^ anion in the experimental geometry broadened by Lorentzian functions with a full width at half max of 0.7 cm^−1^ (red). The blue lines are the *Γ*-point Raman active frequencies for the PW-DFT optimized *P*3̄*m*1 structure. All spectra were normalized to their maximum of absorbance.

The unit-cell of 1 is shown in Fig. 2. Similar to the alkali salts of [MF_6_]^2−^ (M = Tc, Re), parameters such as identical Re–F bond length (1.958(5) Å) and relative F–Re–F angles 90° and 180° are well represented in the Raman spectrum of 1. Here, the Raman spectrum of 1 (Fig. 5) exhibits three unique bands with central frequencies 617, 532, and 214 cm^−1^. The DFT predicted spectrum of a gas phase [ReF_6_]^2−^ anion (in the crystalline geometry) provides a reasonable description of these bands, demonstrating that they primarily correspond to the 6 well-known Raman active vibrational modes of an octahedron.^34^ The 617 cm^−1^ band is the A_1g_ symmetric stretch of the Re–F bonds. The 532 cm^−1^ band is the doubly degenerate E_g_ asymmetric stretch of the Re–F bonds. DFT predictions of these 3 modes for both gas phase [ReF_6_]^2−^ and the *P*3̄*m*1 PW-DFT structure described previously are within 10 cm^−1^ of the measured values.

The gas phase [ReF_6_]^2−^ simulation assigns the 214 cm^−1^ band to the triply degenerate F_2g_ octahedral Re–F bending modes which spilt by 7 cm^−1^ due to the slight deviation (>2°) from octahedral symmetry in the crystal structure. However, the gas phase frequencies are too low and cannot explain the lower frequency shoulder seen experimentally. PW-DFT predicts two of the F_2g_ modes at 206 and the other at 229 cm^−1^. The cause of this splitting is a coupling with rocking/twisting motions of the [NH_4_]^+^ cations, but the inferred lineshape caused by the splitting seems in good agreement with the experimental spectrum. The PW-DFT simulations identify 5 additional modes within the span of the 214 cm^−1^ band: 2E_g_ [NH_4_]^+^ twisting modes at 181 cm^−1^, an A_1g_ [NH_4_]^+^ translation mode at 180 cm^−1^, and 2E_g_ [NH_4_]^+^ translation modes at 165 cm^−1^. Although Raman intensities could not be calculated for the spin-polarized PW-DFT simulations, these center-of-mass motions are presumed to be weak and comprise the lower frequency shoulder of the 214 cm^−1^ band. The PW-DFT calculations also predict 5 modes between 1401 and 1678 cm^−1^, a range typically associated with *ν*_4_ and *ν*_2_ [NH_4_]^+^ bending/librational modes.^35^ There are also 4 Raman active modes between 3322–3445 cm^−1^ corresponding to stretching modes of the N–H bonds.^35,36^

### Magnetic properties of 1

**Fig. 6:**
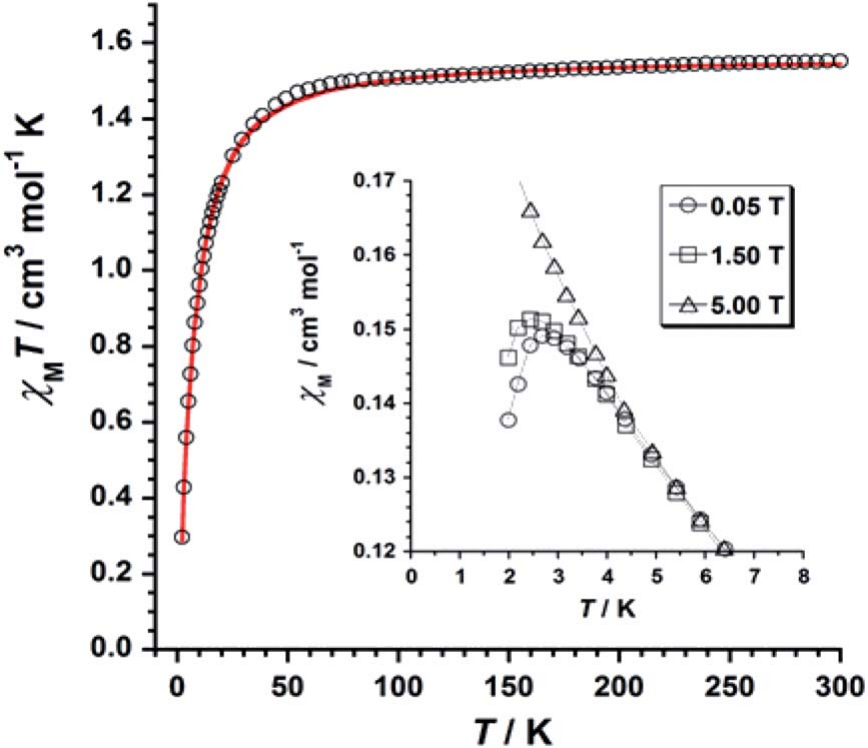
*χ*_M_ *T versus T* plot obtained for 1. The solid red line represents the best-fit of the experimental data. The inset shows the temperature dependence of the magnetic susceptibility at the indicated DC fields for 1.

The *χ*_M_*T versus T* plot (*χ*_M_ being the molar magnetic susceptibility per Re^IV^ ion) is given in Fig. 6. The *χ*_M_*T* value of 1.55 cm^3^ mol^−1^ K observed at *T* = 300 K is as expected for one Re(iv) ion with *S* = 3/2 and *g* ≈ 1.7–1.9.^19^

Upon cooling, *χ*_M_*T* decreases gradually with decreasing temperature, and much faster at approximately 50 K, reaching a minimum value of 0.29 cm^3^ mol^−1^ K at 2.0 K. The decrease of *χ*_M_*T* observed for 1 is likely due to the presence of intermolecular antiferromagnetic interactions and zero-field splitting (ZFS) effects of the Re(iv) ion.^19^ The *χ*_M_*versus T* plot obtained under small DC fields exhibits a maximum at *ca.* 2.9 K indicating the presence of antiferromagnetic exchanges between neighboring Re(iv) ions at very low temperature (see inset in Fig. 6), which would take place in the crystal lattice of 1 through short F⋯F interactions (Fig. 3). Nevertheless, this maximum wanes when higher magnetic fields are applied, thus suggesting the occurrence of a field-induced antiferromagnetic-to-paramagnetic ordering transition for 1 at *T*_N_ ≈ 2.9 K (Fig. S2†), which is a magnetic behavior typical of metamagnetism phenomenon, as previously observed in systems based on Re(iv) ion.^37^

Taking the crystal structure of 1 into consideration, where short Re–F⋯F–Re interactions are observed (Fig. 3), we have used the Hamiltonian of eqn (1), and its derived theoretical expression for the magnetic susceptibility including a *θ* term to account for the intermolecular interactions,^19,37–39^ in order to analyze the magnetic behavior of 1.

1 *Ĥ* = *D*[(*Ŝ*_Z_)^2^ − *S*(*S* + 1)/3] + *gβHŜ*

Best least-squares fits of the experimental data in the 2–300 K temperature range afforded the magnetic parameters: *D* = +11.4(2) cm^−1^, *g* = 1.84(2) and *θ* = −3.1(1) K with *R* = 4.9 × 10^−5^ for 1 {*R* being the agreement factor defined as *Σ*_i_[(*χ*_M_*T*)^obs^_i_ − (*χ*_M_*T*)^calcd^_i_]^2^/[(*χ*_M_*T*)^obs^_i_]^2^}.

The theoretical curve (red solid line in Fig. 6) matches the experimental data well in the whole temperature range. The *g* and *D* values calculated for 1 are in agreement with those previously reported for similar Re(iv) compounds.^19^ The negative *θ* value obtained from the fit implies the occurrence of significant antiferromagnetic exchange between Re(iv) ions through short Re–F⋯F–Re (Fig. 3) intermolecular interactions.

**Fig. 7:**
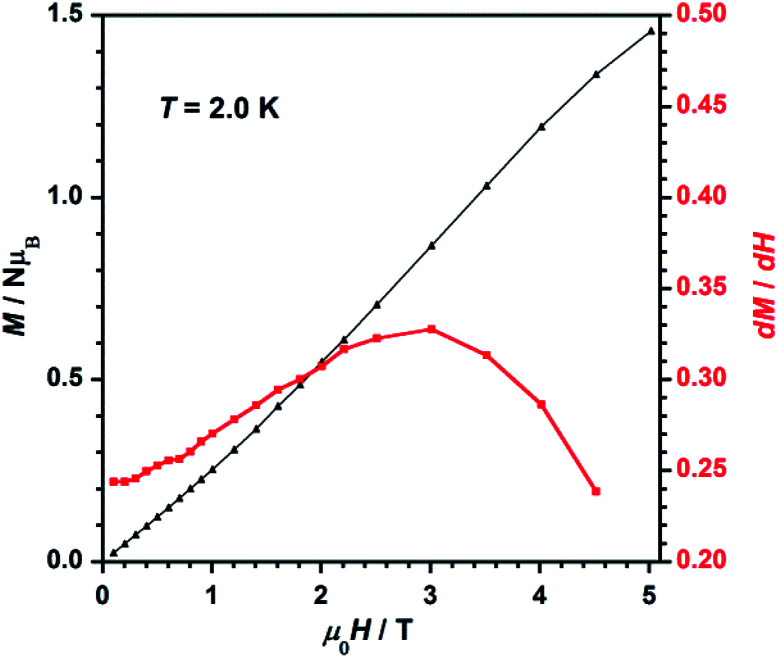
Dependence of the applied DC magnetic field on the magnetization (*M*, black-triangles) and on the derivative of *M* (d*M*/d*H*, red-squares) for 1. The solid lines are guides to the eye only.

The field dependence of magnetization, or *M versus H* plot (*M* being the magnetization per Re(iv) ion and *H* the applied DC magnetic field), and its d*M*/d*H* curve measured at 2.0 K are shown in Fig. 7. *M* values increase with applied field first linearly and somewhat faster at high fields when a smooth inflexion is reached. A critical field (*H*_c_) of *ca.* 3.0 T is detected through a maximum in the d*M*/d*H versus H* curve (Fig. 7), indicating that higher DC magnetic fields (*H* > *H*_c_) overcome the antiferromagnetic interactions observed between metal ions in compound 1. Hence, these features support the occurrence of metamagnetic behavior in 1.^37^

**Fig. 8:**
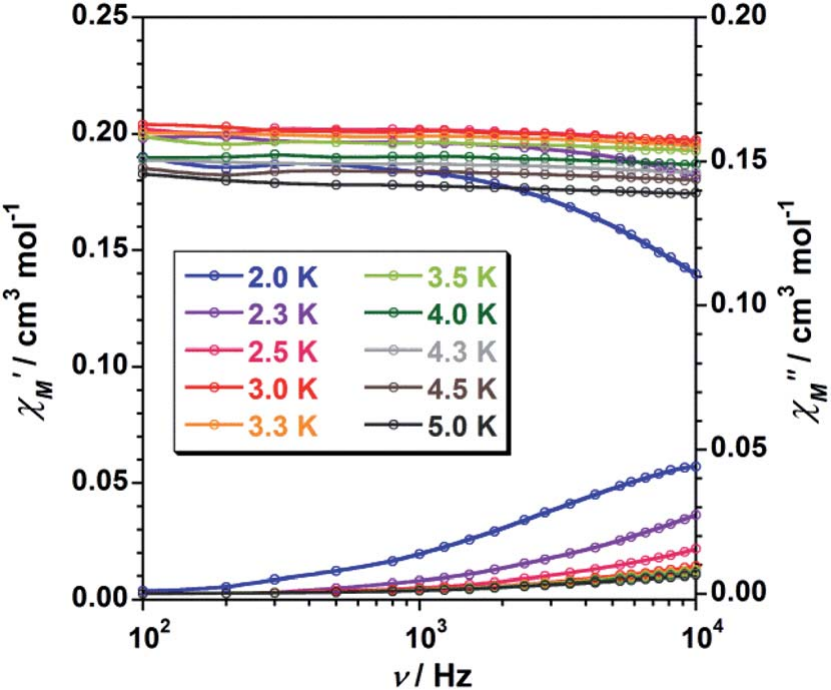
Frequency dependence of the in-phase 
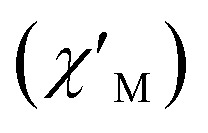
 and out-of-phase 
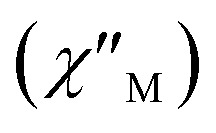
 ac magnetic susceptibility signals for compound 1. Measurements performed at different temperatures and under a dc field of 1000 G.

To study further the magnetic properties of the (NH_4_)_2_[ReF_6_] salt, AC magnetic susceptibility measurements were performed on a sample of this system in the temperature range of 2–5 K and under 0 and 1000 G DC fields oscillating at several frequencies (Fig. 8 and S3†). Interestingly, compound 1 exhibits incipient frequency-dependent out-of-phase AC signals 
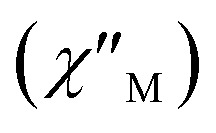
 at very low temperatures (Fig. 8 and S3†), which is indicative of a system with slow relaxation of magnetization. A small dependence on the external DC field is observed in these AC signals for 1 (Fig. S4†). This phenomenon, that is typical of single-ion and single-molecule magnets, has been previously observed in similar Re(iv) complexes^40–42^ but this is the first time that it is observed in a Re(iv) system also showing metamagnetic behavior.

**Fig. 9:**
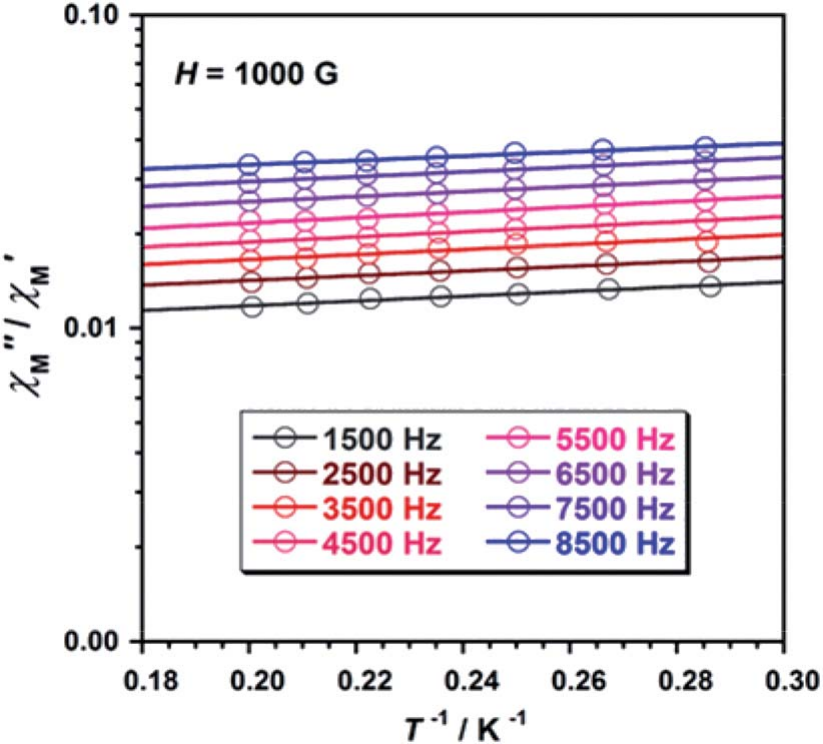
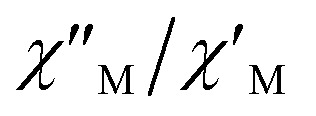
 *versus* 1/*T* plot for 1 at different frequencies (1500–8500 Hz range) and *H*_DC_ = 1000 G. The solid lines are the best-fit curves (see text).

Unfortunately, the expected 
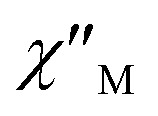
 maxima for 1 are not detected above 2.0 K (Fig. 8 and S3†), given that they would occur at lower temperatures or higher frequencies than those available in our device. So that, it is not possible to quantify magnetic parameters such as the anisotropy energy barrier to magnetization reorientation (*E*_a_) and the preexponential factor (*τ*_o_) for 1 through the Arrhenius expression. Nevertheless, these magnetic parameters could be roughly evaluated by means of the 
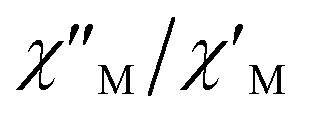
*versus* 1/*T* plot (Fig. 9), the 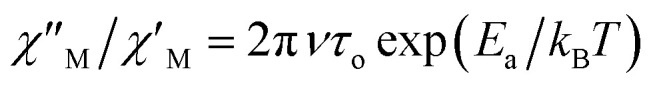 expression, and by assuming only a single relaxation time,^43^ as previously done.^42^ In the case of compound 1, the thus estimated value of *E*_a_ parameter is 1.80 K (1.25 cm^−1^) and that of *τ*_o_ is 5.3 × 10^−7^ s, which were obtained in presence of an external DC field (*H*_DC_ = 1000 G) (Fig. 9). These results are in good agreement with those found in the literature for some mono- and polynuclear Re(iv) systems showing slow relaxation of the magnetization.^16,40,41^ The presence of the NH_4_^+^ cation allows intermolecular halogen⋯halogen interactions that directly affect to the magnetic properties, leading to the magnetic behaviors observed in 1. However, the bulky PPh_4_^+^ cation in the previously reported (PPh_4_)_2_[ReF_6_] salt causes the magnetic dilution of the hexahalorhenate(iv) units (because of the great F⋯F separation that is generated) and magnetic interactions among neighboring paramagnetic [ReF_6_]^2−^ anions are precluded.^9^

## Conclusions

For the first time, the (NH_4_)_2_[ReF_6_] (1) salt was characterized by X-ray diffraction, IR and Raman spectroscopies, DC and AC magnetic susceptibility measurements, and computational methods. 1 crystallizes in the trigonal space group *P*3̄*m*1 and is isostructural to its Tc homologue. The structure of 1 exhibits intermolecular F⋯F interactions (*via* Re–F⋯F–Re) and N⋯F interactions (*via* Re–F⋯N⋯F–Re). In 1, splitting of the Raman peaks (Re–F; F–Re–F) are correlated with the site symmetries of [ReF_6_]^2−^ anion. The study of the magnetic properties, through DC and AC magnetic susceptibility measurements, reveals that metamagnetism and slow relaxation of magnetization phenomena coexist in 1, which is unusual in the molecular systems based on paramagnetic 5d metal ions reported so far. Hence, 1 exhibits a magnetic behaviour that is between that exhibited by the previously reported (PPh_4_)_2_[ReF_6_] single-ion magnet and the typical antiferromagnetic ordering of [ReX_6_]^2−^ salts exhibiting short intermolecular X⋯X interactions. The magnetic chemistry of technetium is underdeveloped and magnetic measurements on [TcF_6_]^2−^ species (4d^3^) have not been performed yet. Work on the preparation and magnetic characterisation of [TcF_6_]^2−^ species is under progress and results will be reported in due course.

## Experimental section

### Materials and reagents

All chemicals and reagents (≥99% purity) were obtained commercially from Sigma Aldrich® and used without any further purification. This work was performed in a well-ventilated fume hood due to the corrosive nature of molten ammonium bifluoride and evolution of gaseous hydrogen halides. The starting material, (NH_4_)_2_[ReBr_6_], was prepared from the reduction of NH_4_[ReO_4_] with H_3_PO_2_ in the presence of NH_4_Br and concentrated HBr as described in the literature.^44^

### Preparation and crystallization of (NH_4_)_2_[ReF_6_] (1)

The (NH_4_)_2_[ReF_6_] salt was prepared from the treatment of (NH_4_)_2_[ReBr_6_] (1.0 g, 1.42 mmol) with excess ammonium bifluoride (0.81 g, 14.2 mmol) at 300 °C under air. Upon cooling, the resulted solid cake was washed with H_2_O/MeOH and dissolved in warm H_2_O for crystallization. The resulting pink 1 was recrystallized in H_2_O and colorless crystals of 1 were obtained within a week. Yield: 300 mg, 60%. Anal. calcd for H_8_N_2_F_6_Re (1): H, 2.4; N, 8.3. Found: H, 2.5; N, 8.4. Raman (cm^−1^): Re–F: 617, 532 and 214. IR (cm^−1^): 472.63, 1000.87, 1078, 1123.94, 1403.89, 2848.8, 2921.8, 3234.59 and 3350.54.

### Physical measurements

Raman spectra were recorded on a HORIBA Jobin Yvon T64000 triple-grating spectrometer operating in subtractive mode. The spectra were collected from pure single crystals at room temperature using the 514.5 nm line of a Kr/Ar laser, with a total of 30 mW incident on the sample. Variable-temperature, solid-state (DC and AC) magnetic susceptibility data down to 2.0 K were collected on a Quantum Design MPMS-XL SQUID magnetometer equipped with a 5 T DC magnet.

### Single crystal XRD data collection and structure refinement

X-ray diffraction data were collected on a Bruker Apex II instrument with Mo-Kα radiation (*λ* = 0.71073 Å) and equipped with an Oxford nitrogen cryostream. The crystal was mounted under Paratone® on a glass fiber; data processing was performed using the Apex III suite software. Structural solution (direct methods) and refinements were completed using SHELXS and SHELX2014 software. Hydrogen atom positions were calculated using the “riding model” option of SHELXL software. The nitrogen atoms of the ammonium ions structure were calculated without the corresponding hydrogen. Crystal parameters and refinement results are summarized in Table 2. The graphical manipulations were performed using Mercury crystal programs.

**Table 2:** Crystal data and refinement parameters for (NH_4_)_2_[ReF_6_] (1)

	(NH_4_)_2_[ReF_6_]
Empirical formula	F_6_N_2_Re
Formula weight	*328.22*
Crystal system	Trigonal
Space group	*P*3̄*m*1
*a* (Å)	6.015(1)
*b* (Å)	6.015(1)
*c* (Å)	4.680(1)
*V* (Å^3^)	146.64(6)
*Z*	1
*ρ* calc (mg m^−3^)	3.71665
*μ* (mm^−1^)	20.764
Reflections collected	1922
Data/restraints/parameters	182/0/12
Goodness-of-fit	1.139
*R*_1_ indices [*I* > 2*σ*(*I*)]	0.0282
w*R*_2_ indices [*I* > 2*σ*(*I*)]	0.0764

### Simulations

Density functional theory (DFT) calculations^45,46^ were performed using the PBE^47^ generalized gradient approximation (GGA) functional. Molecular (*in vacuo*) simulations were performed with the Q-Chem 4.0 package^48^ using the def2-TZVP^49,50^ basis set with small core effective core potentials (ECPs), a 10^−14^*E*_h_ integral cutoff, a 10^−9^*E*_h_ (hartree) energy criterion, and a 10^−4^*E*_h_/Å force criterion. Plane-wave (PW-DFT) calculations on the periodic crystal structure of the solid were performed with CASTEP^51^ using the NCP17 pseudopotentials (Re: 5p^6^6s^2^5d^5^), a 4 × 4 × 4 *Γ*-centered *k*-point grid, a 600 eV plane-wave cutoff, a 10^−12^ eV energy criterion, and a 10^−4^ eV Å^−1^ force criterion. Initial structures were taken from the experimental single crystal structure with hydrogens added manually. For all PW-DFT structural optimizations, the atoms were allowed to move but the unit cell dimensions were fixed at the experimental values in Table 2. Localized orbital bonding analysis (LOBA)^52^ used the Pipek–Mezey^53^ localized orbitals. Vibrational analysis was performed by linear response for molecular frequencies and by finite difference for *Γ*-point plane-wave frequencies and molecular intensities.^54^ The PW-DFT phonon calculations were on a single unit cell that has only one *S* = 3/2 Re atom, therefore the Re magnetic moments were effectively aligned ferromagnetically.

## Funding sources

This material is based upon work supported by the Department of Energy National Nuclear Security Administration through the Nuclear Science and Security Consortium under Award Number(s) DE-NA0003180. Portions of this work were performed at HPCAT (Sector 16), Advanced Photon Source (APS), Argonne National Laboratory. HPCAT operations are supported by DOE-NNSA's Office of Experimental Sciences. The Advanced Photon Source is a U.S. Department of Energy (DOE) Office of Science User Facility operated for the DOE Office of Science by Argonne National Laboratory under Contract No. DE-AC02-06CH11357. The Spanish Ministry of Science, Innovation and Universities has also supported part of this work through the projects PID2019-109735GB-I00 and MDM-2015-0538 (Excellence Unit “María de Maeztu”).

## Conflicts of interest

The authors declare no conflict of interest.

## Supplementary Material

RA-011-D0RA10325J-s001

RA-011-D0RA10325J-s002
