# Supplementary material for: Coexistence of metamagnetism and slow relaxation of magnetization in ammonium hexafluoridorhenate
Source: RSC Adv. 2021 Feb 3;11(11):6353–60. doi: 10.1039/d0ra10325j (PMC8694846; doi:10.1039/d0ra10325j)
Supplement: RA-011-D0RA10325J-s001 [file RA-011-D0RA10325J-s001.pdf]

Electronic Supplementary Material (ESI) for RSC Advances.
This journal is © The Royal Society of Chemistry 2021

Coexistence of Metamagnetism and Slow Relaxation of Magnetization in Ammonium Hexafluoridorhenate

James Louis-Jean,*[a] Samundeeswari M. Balasekaran,[a] Keith V. Lawler,‡[a] Adrián Sanchis-Perucho,‡[b] José Martínez-Lillo,*[b] Dean Smith,[c] Paul M. Forster,[a] Ashkan Salamat,[c] and Frederic Poineau*[a]

[a.] *Department of Chemistry and Biochemistry, University of Nevada Las Vegas, 4505 South Maryland Parkway, Las Vegas, Nevada, 89154-4003, United States. E-mail: louisjea@unlv.nevada.edu (JLJ); poineauf@unlv.nevada.edu (FP)*
[b.] *Instituto de Ciencia Molecular (ICMol)/Departament de Química Inorgànica, Universitat de València, Catedrático José Beltrán 2, 46980 Paterna, València, Spain. E-mail: f.jose.martinez@uv.es (JML)*
[c.] *Department of Physics and Astronomy, University of Nevada Las Vegas, 4505 South Maryland Parkway, Las Vegas, Nevada, 89154-4003, United States.*
‡ These authors contributed equally to this work.

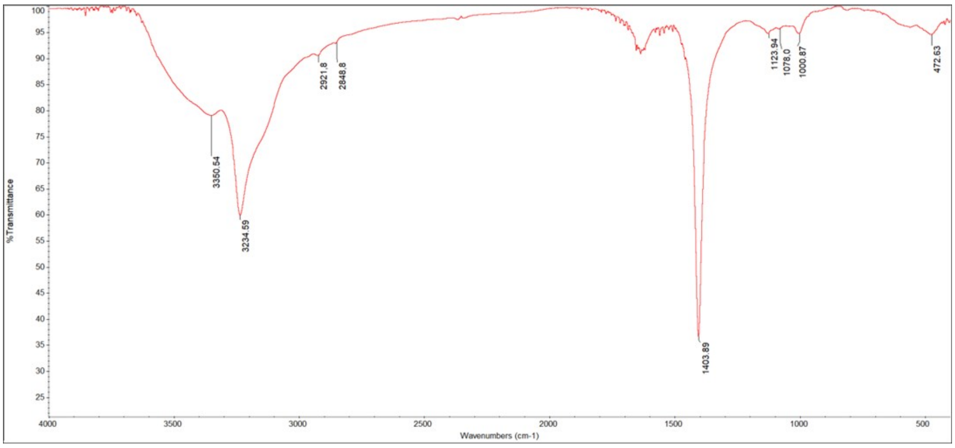

Fig. S1. Infrared spectrum (IR) for compound **1**.

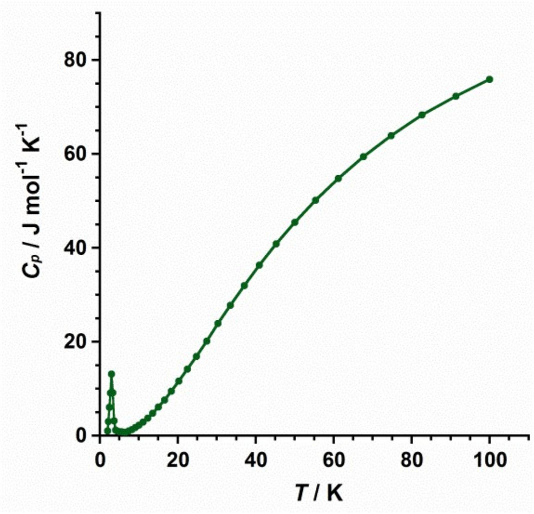

Fig. S2. Heat capacity (C_p) *versus* temperature plot for compound **1**.

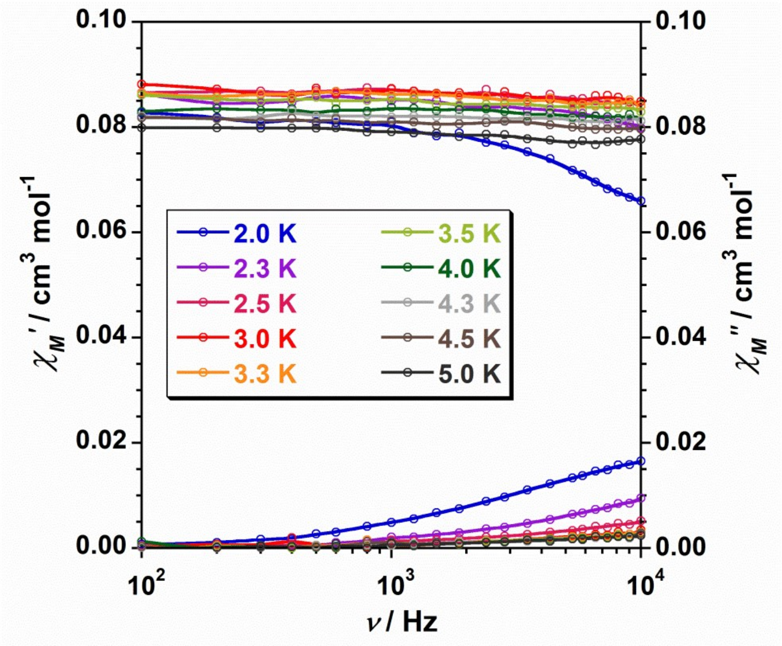

Fig. S3. Frequency dependence of the in-phase (χ_M') and out-of-phase (χ_M'') ac magnetic susceptibility signals for compound **1**. Measurements performed at different temperatures and H_{dc} = 0 G.

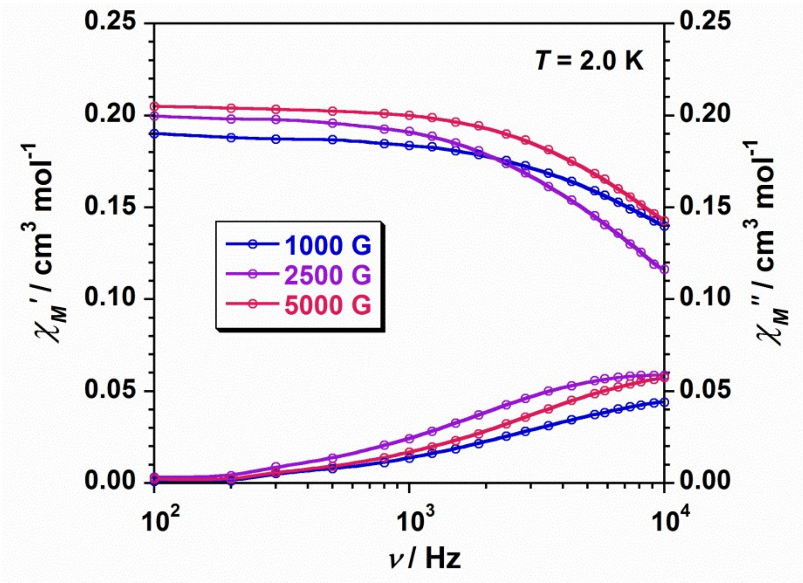

Fig. S4. Frequency dependence of the in-phase (χ_M') and out-of-phase (χ_M'') ac magnetic susceptibility signals for compound **1**. Measurements performed at different dc fields and *T* = 2.0 K.
